# The Effect of Psychological Contract Combined With Stress and Health on Employees’ Management Behavior

**DOI:** 10.3389/fpsyg.2021.667302

**Published:** 2021-06-10

**Authors:** Yueyuan Cheng

**Affiliations:** Department of Foreign Languages, Zunyi Medical University Zhuhai Campus, Zhuhai, China

**Keywords:** psychological contract, employee management, stress and health, FMRI, management behavior

## Abstract

In order to effectively manage employees and improve their work enthusiasm, based on the theoretical basis of project team members’ psychological contract, the *status quo* of employees’ psychological contract, stress and health status are investigated and analyzed. The differences of employees’ psychological contract are analyzed through the questionnaire. A total of 400 questionnaires are distributed and 365 are returned. The method of one-way ANOVA is used to study the psychological contract status of project members from four aspects: gender, education background, position level, and working years, and obtain the results of difference analysis in four aspects. Functional magnetic resonance imaging (FMRI) is used to evaluate the work stress of employees. The results show that gender, education background, position level and working years have different effects on employees’ psychological contract. Moreover, if employees continue to bear high-intensity work stress, a series of psychological and physical health problems will appear, such as difficulty in concentration. For enterprises, the poor psychological and physical conditions of employees will make the overall work inefficient and the working atmosphere dull. Therefore, enterprises should control employees’ work stress within a reasonable range, and should not blindly impose high-intensity work stress on employees. This exploration demonstrates the way to realize the psychological contract construction of project team members, and targeted employee management countermeasures are put forward.

## Introduction

Work stress has become an inevitable universal problem in people’s work and life. Excessive work stress will seriously affect the quality of work and life of employees, and indirectly lead to the decrease of the company’s or organization’s benefit (Ihlström et al., 2017; Mäcken, 2019). Work stress has two sides. For most people, work stress has both positive and negative aspects (Liu et al., 2019). For example, when people are faced with difficult work, they will feel a sense of excitement and motivation, and feel a certain degree of threat and uneasiness at the same time. The promotion of the position will also have two effects. On the one hand, in the face of a new position, the party concerned will have a fear that they do not understand the situation, worry about the disharmony of interpersonal relationship in the new working environment, and whether they are competent for the position. On the other hand, the acquisition of new positions will make them eager to carry out their work in order to expect more new challenges, rewards and satisfaction (Unanue et al., 2017; Mullen et al., 2018). In this case, new and unknown working conditions create favorable stresses. Then, the existence of work stress is inevitable. There is no doubt that there is no working and living environment without any stress. Psychological contract is a kind of tacit agreement between employees and enterprises, which will have an important impact on both employees and enterprises (Poisat et al., 2018). Therefore, research on employee management will be carried out from the aspects of psychological contract, stress and health.

Many scholars have studied the related aspects of employee management. Li et al. (2016) proposed a multi-level model in which the relationships among competitive climate, organizational identity, job performance, emotional commitment and psychological contract violation were obtained from a sample of hotel employees. The results show that the competitive atmosphere at the organizational level can adjust the personal behavior violation as well as the impact of personal behavior violation on hotel employee role performance (Li et al., 2016). In terms of productivity and performance, turnover is a costly phenomenon. Flores et al. (2019) investigated whether emotional exhaustion, organizational cynicism and breach of psychological contract preceded the purpose of leaving. To this end, 201 operators of auto parts manufacturers were studied quantitatively, empirically, and horizontally. It is found that there is a very close relationship between employees’ leaving and their psychological contract (Flores et al., 2019). Haski-Leventhal et al. (2020) used the psychological contract theory to compare the expectations and results of students, universities and non-profit organizations, and proposed a model of volunteer service benefits for all three stakeholder groups. The results show that training, preparation, and management expectations have the potential to create positive benefits for all (Haski-Leventhal et al., 2020). Hong and Kim (2016) studied the impact of psychological contract violation on job satisfaction and turnover intention of catering employees in luxury hotels. On the basis of 280 samples for empirical research, the reliability and fitting degree of the research model were reviewed, and four hypotheses were verified. The results show that the relationship contract breach has a significant negative impact on job satisfaction, and job satisfaction has a significant negative impact on turnover intention (Hong and Kim, 2016). Van Hootegem and De Witte (2019) explored whether job insecurity is related to low-level information seeking, feedback seeking and help seeking behaviors, and whether these relationships are regulated by professional self-efficacy and psychological contract. The results show that violation of psychological contract can only mediate job insecurity. Due to the decrease of professional self-efficacy and the increase of psychological contract violation, job insecurity will reduce the possibility of employees to participate in informal learning, and thus become more uncompetitive in the increasingly unstable working environment (Van Hootegem and De Witte, 2019).

The above description shows that the current research on the relationship between psychological contract and employees is mostly about the impact of psychological contract on employee turnover. In order to effectively manage employees and improve their work enthusiasm, employee management has been studied from the perspective of psychological contract, stress and health. It is expected that good research results can be obtained in the relevant direction, which will provide new ideas for the enterprise to choose employee management plan. After the basic theoretical basis of psychological contract is analyzed, the idea of updating traditional management concept is put forward. The test method combining theory and empirical research is adopted. Through the questionnaire, the empirical study is carried out to analyze the impact of employee management on the construction of psychological contract. Next, the functional magnetic resonance imaging (FMRI) of employees with different work stresses is studied, the differences in neural activities among different employees with different working stresses are explored, and the inspiration to managers in personnel management is extended.

## Materials and Methods

### Psychological Contract

Psychological contract is currently recognized as self-interest, which is a corresponding cooperation between individuals and enterprises (Solinger et al., 2016; Rousseau et al., 2018; Soares and Mosquera, 2019). In the psychological contract, it emphasizes that employees should try their best to create their own value in the enterprise and help the enterprise develop better, and the enterprise should meet the material and psychological needs of employees (Karagonlar et al., 2016; Newaz et al., 2019). Although psychological contract does not appear in written form in the legal relationship between employees and enterprises, psychological contract has become an important tacit agreement between enterprises and employees to maintain the legal relationship between enterprises and employees.

Between enterprises and employees, the most important manifestation of psychological contract is employee satisfaction with the enterprise (Lub et al., 2016). If the employees can satisfy all kinds of material and psychological needs brought by enterprises, there will be a good relationship between enterprises and employees. Only in this way can enterprises develop with higher quality and employees get better promotion. According to the psychological contract, employees’ needs for the enterprise can be divided into seven parts: environment, task, ownership, reward, value, development, and promotion. The above seven parts completely summarize all the needs of employees for the ideal enterprise and the important guarantee of employees’ work. In the enterprise aspect, the enterprise needs the employee to have the more outstanding work ability, has the high loyalty to the enterprise, can put forward the constructive opinion, abides by the company’s rules and regulations and so on. Therefore, in the relationship between employees and enterprises, employees are the subject of psychological contract (Gupta et al., 2016; Costa and Neves, 2017; Han et al., 2017).

### The Construction of Employee Psychological Contract

Human resource is the most valuable resource in project management. To improve the level of human resource management and form an effective psychological contract among project employees is an important problem that project managers must deeply understand and try to solve. Project managers should strive to improve the efficiency of personnel introduction, reduce costs, ensure the acquisition of human resources required by the project, optimize the human resource structure of project organization, and enhance the adaptability of the project team.

The organization should form a mutual trust and mutual respect labor relationship with employees in the process of project management. In the process of achieving the objectives, the organization should also try to meet the reasonable requirements of employees, closely link the fate of the organization and employees, so that the symbiotic relationship between the organization and the project employee can be fully reflected. The purpose of project employee is to pursue good career development, and they attach importance to personal career planning. When the project employee work hard for their own survival and career, they have recognized the value orientation of the organization in a sense. If the organization can create a good working atmosphere to achieve people’s success, and know one’s subordinates well enough to assign them jobs commensurate with their abilities, the project employee can provide services for the project team at ease. On the contrary, without a good system and scientific human resource planning, the project employee cannot see the development direction of their personal career. Even if they are given high salary, it is difficult to maintain the stability of the project team. To formulate incentive measures for project employees, it is necessary to start from meeting the needs of project employees, and formulate feasible incentive objectives and measures, so as to achieve the expected incentive effect.

### Study on the Relationship Between Stress and Employee Health

Sub-health refers to a critical state of non-disease and non-health, which is a secondary health state between health and disease. Therefore, it is also called “secondary health,” “third state,” “intermediate state,” “wandering state,” and “gray state” (Xu et al., 2017; Zhao et al., 2017). The long-term sub-health status of employees will have a very serious impact on their physical and psychological health and other aspects (Shen et al., 2018).

White-collar workers are the main sub-health population (Xu et al., 2019). Due to the stress of work and life, white-collar workers suffer from physical and psychological fatigue. Therefore, white-collar workers are the main sub-health population (Shin, 2018a). The social life rhythm of white-collar class is fast, and the psychological stress is big. The complex urban life and interpersonal relationships, unavoidable risks, unexpected setbacks, deterioration of environmental quality, irregular life, especially smoking, drinking, overeating, and lack of necessary exercise, make many people fall into sub-health state (Feng et al., 2016; Shin, 2018b).

According to the clinical manifestations of sub-health population, in *Sub-health Chinese Medicine Clinical Guidelines*, sub-health population is divided into the following three categories: (1) psychological sub-health: it is mainly reflected in tension, irritability, anxiety, restlessness, inattention, short-term memory loss, panic and other mental and psychological symptoms; (2) physical sub-health: it is mainly reflected in muscle soreness, sleep disorders, fatigue and other physical symptoms; (3) social communication sub-health: it is mainly reflected in the social adaptability such as inability to deal with interpersonal relationships, reducing the frequency of social interaction, and difficult to integrate into social roles.

FMRI detection technology is used to realize real-time monitoring of human brain neural activity, which can directly reflect the activity status of various functional brain regions of the human brain, and plays an important role in the measurement and control of employees’ sub-health status and work stress (Macefield et al., 2013). Resting-state FMRI is used to compare the structural and functional differences in brain regions between employees with high work stress and in control group.

### Analysis of Employee Health Status Based on FMRI

Regional Homogeneity (ReHo) method is used to process FMRI data. Now, f(M,N,O,T) is used to represent a FMRI data. Where M is the number of rows, N is the number of columns, O is the number of layers, T is the number of time points (the length of the time series) of each voxel, and the voxel number of the dataset is MxNxO. For a voxel V(m,n,o) (1 < m < M, 1 < n < N, 1 < o < O), the ReHo of the voxel time series and the voxel of its nearest neighborhood K (generally *K* = 6, 18, 26) is calculated as follows:

(1) The time series of K + 1 voxels is expressed as a matrix X with the size of Tx (K + 1). Where X (i, j) denotes the i-th time point of j-th voxel;
(2) The element in column j is replaced by the rank of the element in the column where it is located (i.e., the ordinal number of the size of the value of the element in column j) to obtain the matrix R with the size of Tx(K + 1). Where R (i, j) denotes the rank of the i-th time point of j-th voxel;
(3) Kendall harmony coefficient measures the similarity of multiple voxel time series. The larger the value, the more similar the time series. When ReHo is used for data analysis, the ReHo value of each voxel and its neighborhood voxel time series will usually be calculated to get a ReHo statistical map of the whole brain.
(4) The ReHo of each voxel is divided by the average ReHo value of the whole brain to standardize. The ReHo value of each voxel after standardization should be around 1.
(5) The statistical graph of ReHo is smoothed.

### Experimental Design

In order to test the influence of the optimization of employee management strategy on the construction of employee psychological contract and the regulation of stress and health, a combination of theoretical and empirical test method is adopted. On the basis of combing the relevant theories, interviews and questionnaires are used to analyze the collected feedback information and try to make statistical analysis in a quantitative way. The corresponding questionnaire implementation procedures, scoring methods, standardized score interpretation and other data are developed, and the relevant questionnaire investigators are trained to ensure that the questionnaire has high reliability and validity. A questionnaire survey is conducted among employees of an Internet company in Hangzhou. The questionnaire is distributed and collected on site.

Questionnaires are sent out to analyze the differences of employees’ psychological contract. A total of 400 questionnaires are distributed and 365 questionnaires are recovered, of which 310 are valid. All employees are divided into high stress group and control group. The experimental method is resting-state FMRI. All subjects in the resting state of brain scan data are collected and comparative analysis is carried out between groups. It needs to be determined that the subject does not have a confined space phobia. The subjects are given a simple explanation of the experimental principle, especially the precautions during the scanning process. Almost all the subjects undergo MRI scan for the first time. In order to eliminate the subjects’ doubts about the safety (radiation) of MRI, it is necessary to explain the basic principle of MRI. The subjects are told not to engage in any specific thinking activities during the whole process of resting state scanning, and they do not need to complete any operation. They must keep lying flat, eyes closed, head fixed and awake throughout the whole process. The ReHo map obtained from the previous processing is tested by false discovery rate (FDR) control test on SPM5 software. In the comparison between groups, it is set that the difference is statistically significant when FDR is not more than 0.05.

The questionnaire is divided into three parts. The first part is the basic situation of the employees, including gender, educational background, position level and working years. The second part is the responsibility that the employees think the project team should bear, and the third part is the responsibility that the employees think they should bear. In the second part and the third part, there are 12 questions, as shown in Table 1. The subjects are assessed with Likert scale. 1 point is never promised, 2 points are implied, 3 points are explicitly implied, 4 points are explicitly promised, and 5 points are very clearly promised. Low points represent low organizational responsibility satisfaction, while high points represent high organizational responsibility satisfaction.

**TABLE 1:** Question setting of the questionnaire.

**Serial number**	**The second part**	**The third part**
1	Provide reasonable remuneration	Observe law and discipline
2	Provide comprehensive welfare	Support project team decision making
3	Good professional quality of managers	Accept job change
4	Provide good living conditions	Keep project confidential
5	Provide training opportunities	Strive to improve working ability
6	Offer promotion opportunities	Accept necessary overtime
7	Employees can participate in the decision-making system	Work in the project team at ease
8	Work is fun	Don’t do anything to the detriment of the project team
9	Encourage collaboration between departments	Have team work spirit
10	Smooth communication channels within the organization	Take the initiative to offer suggestions for the project team
11	Care about project employee and their family life	Establish a good relationship with colleagues
12	Respect employees	Work closely with colleagues

## Results

### Demographic Characteristics of the Subjects

**FIGURE 1:**
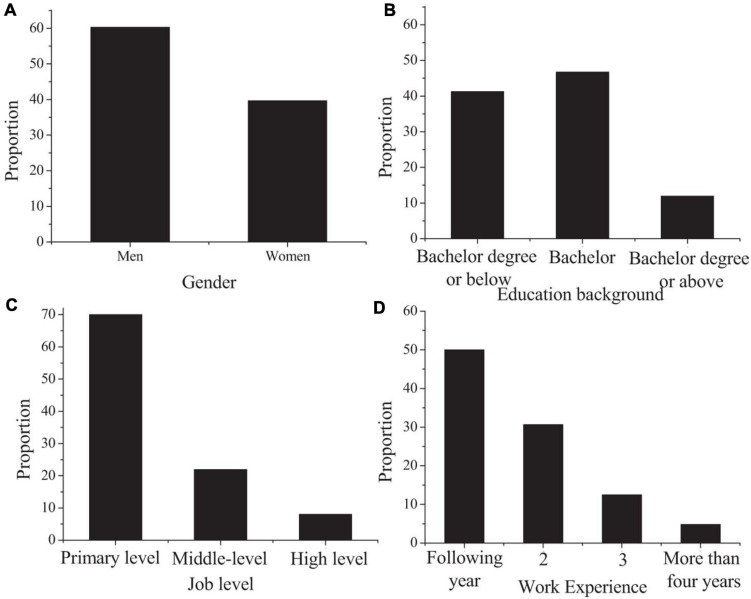
Demographic characteristics of participating researchers (**A:** gender; **B:** educational background; **C:** position level; **D:** working years).

By sorting out the data in the questionnaire, the demographic characteristics as shown in Figure 1 are obtained.

### The Influence of Employee Gender and Psychological Contract

**FIGURE 2:**
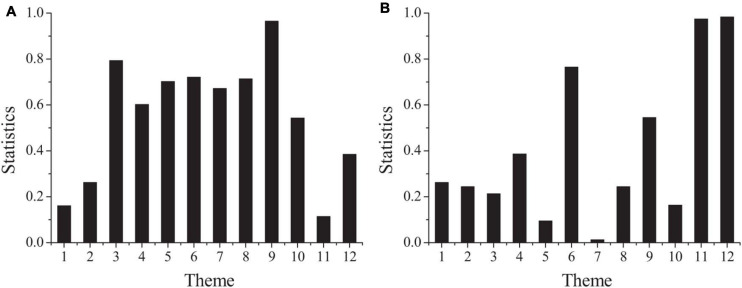
The differences of psychological contract in different genders (**A:** the second part; **B:** the third part).

In order to compare whether there is significant difference in psychological contract between male and female project employees, one-way ANOVA is used to test the difference between male and female project employees. Figure 2 shows the results.

Figure 2 shows that the concomitant probability of striving to improve the working ability and level and to work in the project team with ease is less than the significance level of 0.1, and the concomitant probability of working in the project team with ease is less than 0.05. It shows that there are significant differences between male and female employees in the perception of these two problems. Striving to improve the working ability and level as well as working in the project team with ease belong to the relationship responsibility of employees. It can be seen that there are significant differences in the dimension of relationship responsibility in the organizational responsibility of project employees of different genders. Due to the differences in salary, welfare and social security between male project employees and female project employees, the sense of responsibility of male project employees is higher than that of female project employees on the whole. Therefore, different strategies should be adopted for different gender project employees. For male employees, pay should be emphasized and highlighted, while for female employees, welfare and social security should be emphasized and highlighted. In the process of training and career planning, female employees should be given more education on their work attitude and sense of responsibility.

### The Influence of Employee’s Educational Background and Psychological Contract

**FIGURE 3:**
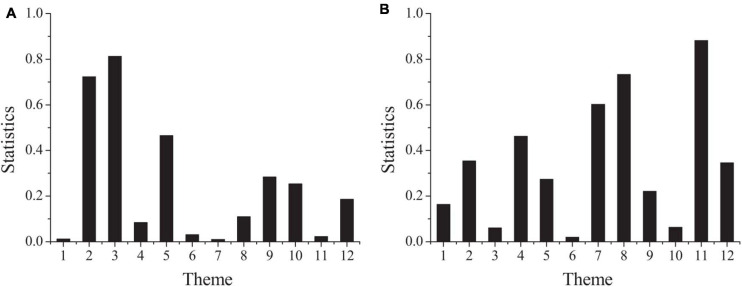
The differences of psychological contract among employees with different educational background (**A:** the second part; **B:** the third part).

In order to understand and master the status of psychological contract of project employees with different educational background, the difference test can be carried out according to the educational background of project employees to compare the significant differences in psychological contract of project employees with different educational background. Figure 3 shows the result.

Figure 3 shows that the concomitant probabilities of questions 1, 4, 6, 7, 8, 11 in the second part and questions 3, 6, and 10 in the third part are all less than 0.05. Among them, the concomitant probabilities of questions 1, 6, 7, 11 in the second part and 6 in the third part are all less than the significance level of 0.1, which indicates that employees with different educational backgrounds have significant differences in the perception of these problems. There are significant differences in organizational responsibility and employee responsibility among project employees with different educational background. The project manager should attach great importance to the guarantee of the material life of the project employees, and provide the employees with satisfactory welfare as far as possible; at the same time, the project manager should establish the management concept of people-oriented and human resources are the most valuable resources of the project team, understand and meet the needs of employees, create a good interpersonal relationship atmosphere, and make the project employees’ life stable and happy, in order to improve the enthusiasm of employees.

### The Influence of Employee Position and Psychological Contract

**FIGURE 4:**
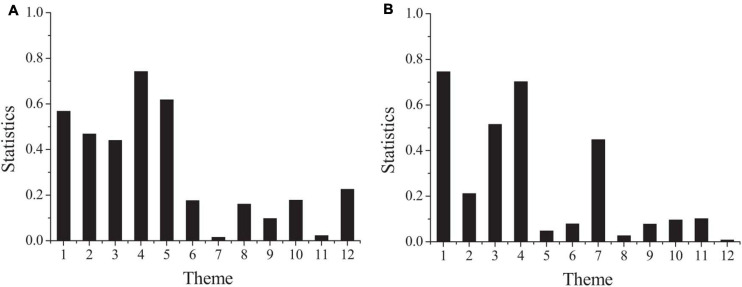
The differences of psychological contract among employees in different positions (**A:** the second part; **B:** the third part).

Through the difference test of the psychological contract status of the grass-roots, middle-level and high-level project employees, whether there are significant differences in the psychological contract of the project employees with different positions can be compared. Figure 4 shows the results.

Figure 4 shows that the concomitant probabilities of questions 7, 9, 11 in the second part and questions 5, 6, 8, 9, 10, and 12 in the third part are all less than 0.1. Among them, the concomitant probabilities of questions 7, 11 in the second part and questions 5, 8, 12 in the third part are less than the significance level of 0.05. The results show that there are obvious differences between project employees of different positions in perception of these problems. It can be seen that there are significant differences in organizational responsibility and employee responsibility among different positions of project employees. For grass-roots employees, it is necessary to try to emphasize the advantages of material life security provided by the organization, such as relatively high remuneration, perfect welfare system, and beautiful working environment, and encourage them to work well in the form of more material incentives. Due to the strong sense of transactional responsibility of the grass-roots project employees, the middle-level project employees and senior project employees should be able to clarify the responsibility and obligation requirements of the grass-roots level as far as possible, and reduce the autonomous work authority and content. The psychological contract between the organization and the individual is two-sided, that is, there exists the psychological contract between the individual and the organization. The important factor of harmonious relationship between organization and employees is psychological contract. The ideal psychological contract is that the enterprise can fully grasp the inner expectations of every employee in the organization and meet them, and every employee in the organization will go all out for the development of the enterprise because the enterprise has realized their expectations.

### The Influence of Employees’ Working Years and Psychological Contract

**FIGURE 5:**
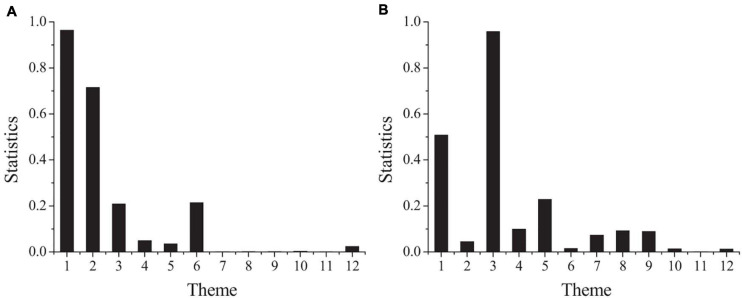
The differences of psychological contract among employees with different working years (**A:** the second part; **B:** the third part).

In order to find out whether there are significant differences in psychological contract among project employees with different working years, the specific results can be obtained through one-way ANOVA. Figure 5 shows the result.

Figure 5 shows that the concomitant probabilities of questions 3, 4, 7, 8, 9, 10 in the second part and 2, 4, 6, 7, 8, 9, 10, 11, and 12 in the third part are all less than the significance level of 0.05. Among them, the concomitant probabilities of questions 3, 4, 5, 7, 8, 9, 10, 11, 12 in the second part and 2, 6, 10, 11, and 12 in the third part are all less than the significance level of 0.1, which indicates that project employees with different working years show significant differences in perception of these problems. The data listed show that there are significant differences in the perception of organizational responsibility and employee responsibility among project employees with different working years. In the management of project employees with working years less than 1 year, the project team should start from improving the quality of management personnel, and win their satisfaction to the organization by building a good management team, and strive to win their trust and loyalty as soon as possible.

Based on the perspective of transaction contract, the company’s psychological contract responsibility should not only include the corresponding material reward, but also guarantee the due spiritual respect, which is embodied in more guarantee of fairness. In the view of employees, in order to meet their recognition of the fairness of transaction contract dimension, they mainly expect that the company can guarantee the fairness of distribution results and the fairness of implementation process, that is, distribution justice and procedural justice. Employees’ inner development goals are the realization of their own growth and self-worth, and the realization of these goals is inseparable from the organization in which the employees live. Employees’ personal growth and the realization of self-worth are inseparable from the growth of enterprises and the realization of enterprise value. Therefore, the managers of enterprises should pay attention to the feedback of the results of employees’ hard work while achieving the organizational goals. They should make the employees fully realize the role of their work in achieving the organizational goals, and let the employees fully realize that the process of achieving the organizational goals is also the process of self-realization of the knowledgeable employees themselves.

### Results of FMRI Study on Employees With Different Stress Levels

The statistical results show that the abnormal situation of decreased nerve activity (ReHo decrease) occurs in the employees of high-stress group under resting state, which involves the central sulcus and parietal cortex on the lateral side of the cerebral hemisphere. Table 2 shows the specific activated brain regions.

**TABLE 2:** ReHo value statistics of high-stress employees.

**Category**	***K*-value**	***Z*-value**	***P*-value after correction**
Angular gyrus of left parietal inferior margin	10	4.87	0.037
Left postcentral gyrus	7	4.63	0.037

The results of image analysis show that the ReHo values in the postcentral gyrus and angular gyrus of parietal inferior margin of the left side of brain are significantly increased in the high-stress group, indicating that the neural activity in this area is stronger at this time. The brain function structure of employees can reflect the work stress, and the high expectation and low work stress level employees have different activation degree in brain area between the employees with low expectation and high working stress. Therefore, the effect of reducing the sense of stress can be achieved by improving the rational expectation of employees based on the expectation level of employees in the control of the work stress factors. The enterprise needs to objectively and comprehensively analyze the situation and realistic resources of employees, help the employees to achieve practical career development, meet the rational expectations of employees as much as possible, guide them to establish their rational expectations, especially in salary and welfare, job promotion, and finally achieve the goal of employee satisfaction.

In view of the work stress of enterprise employees, the first way to alleviate the stress is to strengthen the competency evaluation of enterprise employees, properly allocate the jobs of employees, and reduce the role overload. Through the post competency test, allocating the right employee for the right post will greatly alleviate the work stress perception of enterprise employees. In addition, it is necessary to strengthen the regulation of employees’ perception of work stress and eliminate the concept of emotional perception. Enterprise leaders should timely identify and eliminate employees’ emotional pressure perception, eliminate the general and unreasonable way of thinking, guide employees’ enthusiasm and confidence, and actively participate in the process of employees’ work stress perception, so as to solve their problems.

## Discussion

The study shows that there are some differences in psychological contract performance between male and female project employees. In terms of employee responsibility, male project employees have a higher sense of responsibility than female project employees. Therefore, for project employees with different genders, it is necessary to take different strategies for this situation. For male employees, pay can be emphasized and highlighted. For female employees, it is necessary to emphasize and highlight the part of welfare and social security. The psychological contract performance of project employees with different educational background is different to some extent. Employees with different educational backgrounds attach great importance to the remuneration of the enterprise, and at the same time, it also shows that the project employees pay attention to others’ attitude toward themselves, hoping to be recognized by others. Based on this, the project manager should attach great importance to the protection of the material life of the project employees, and provide the employees with satisfactory welfare as far as possible. In view of the highest average score of the transactional responsibility dimension of the grass-roots project employees, it means that they pay more attention to the transactional responsibility in the organizational responsibility. Therefore, for the management measures for grass-roots employees, it is necessary to emphasize the advantages of material life security provided by the organization, such as relatively high remuneration, perfect welfare system, and beautiful working environment, and encourage them to work well in the form of more material incentives. Material incentive is the most basic factor to mobilize the enthusiasm of employees. If the most basic material needs of the project employees cannot be well met, it is empty talk to mobilize the enthusiasm of the employees. Material incentive is mainly used to meet the material needs of the project employees. The main forms include basic salary, bonus, special contribution award, employee welfare and so on. The establishment of a material incentive mode suitable for project management can well mobilize the enthusiasm of employees. On this basis, the implementation of other incentive methods can also play a better role in incentive. The material incentive can be the performance salary incentive, the special topic reward and other salary incentive ways.

From the research on employee stress and health, the more stressed employees are, the more likely they are to fall into unhealthy emotions. Khamisa et al. (2017) tried to determine whether personal stress was more predictive of burnout, job satisfaction and general health than work stress. The results show that personal stress can better predict job burnout and overall health status than work stress, while work stress can better predict job satisfaction (Khamisa et al., 2017), which is basically consistent with the results of this study. In Mensah’s (2019) research, it is also confirmed that the psychological contract mechanism has an impact on talent management, which may ultimately affect the performance of talented employees (Mensah, 2019; Shen et al., 2019). Based on the existing research, the influence of employee stress and health as a factor on enterprise talent management is included, which supplements the research blank in this field.

When there is a certain gap between employees’ actual income and psychological expectation due to the enterprise’s reasons, it will lead to the violation of psychological contract (Zhou and Wu, 2018; Wu and Wu, 2019). Therefore, first, enterprises should have a reasonable and effective communication mechanism. Communication plays an important role in forming a good contract. When new employees are employed, they should first feel that the enterprise is a big family, which requires honest and in-depth communication between managers and employees (Chen, 2019; Zheng et al., 2020). Through effective communication, managers can understand employees’ inner needs and motivate some employees’ needs. In addition, they need to pay attention to the psychological contract of knowledge workers. Knowledge workers are a special group who pursue the realization of self-worth, so enterprises should try to help them realize their own value.

## Conclusion

The relationship among employee psychological contract, stress and health and employee management has been mainly studied. The results show that the concomitant probabilities of questions 3, 4, 5, 7, 8, 9, 10, 11, and 12 in the second part and questions 2, 6, 10, 11, and 12 in the third part of the questionnaire are less than the significance level of 0.1. It shows that there are obvious differences in the cognition of these problems among employees in different positions. This exploration confirms that the optimization of employee management based on psychological contract can be used as a feasible starting point, but there are still some deficiencies. The impact of the rapid development of information society on employees is increasingly deepening, and employees’ understanding of enterprise psychological contract is bound to have new changes, which will be a new research direction in the future.

## Data Availability Statement

The raw data supporting the conclusions of this article will be made available by the authors, without undue reservation, to any qualified researcher.

## Ethics Statement

The studies involving human participants were reviewed and approved by the Zunyi Medical University Zhuhai Campus Ethics Committee. The patients/participants provided their written informed consent to participate in this study. Written informed consent was obtained from the individual(s) for the publication of any potentially identifiable images or data included in this article.

## Author Contributions

The author confirms being the sole contributor of this work and has approved it for publication.

## Conflict of Interest

The author declares that the research was conducted in the absence of any commercial or financial relationships that could be construed as a potential conflict of interest.
